# Evaluation of *LOXL1* polymorphisms in exfoliation syndrome in a Chinese population

**Published:** 2009-11-14

**Authors:** Ling Chen, Liyun Jia, Ningli Wang, Guangxian Tang, Chun Zhang, Sujie Fan, Wenru Liu, Hailin Meng, Wotan Zeng, Ningpu Liu, Huaizhou Wang, Hongyan Jia

**Affiliations:** 1 Beijing Tongren Eye Center, Beijing Tongren Hospital, Capital Medical University, Beijing Ophthalmology & Visual Science Key Lab, Beijing, China; 2 Department of Ophthalmology, XingTai Eye Hospital, XingTai, China; 3 Peking University Eye Center, Peking University Third Hospital, Beijing, China; 4 Department of Ophthalmology, Handan Eye Hospital, Handan, China; 5 Department of Ophthalmology, Anyang Eye Hospital, Anyang, China; 6 Chinese National Human Genome Center, Beijing, China

## Abstract

**Purpose:**

To evaluate the association profiles of the lysyl oxidase-like 1 (*LOXL1*) gene polymorphisms with exfoliation syndrome in a Chinese population.

**Methods:**

Fifty unrelated patients with exfoliation syndrome and 125 control subjects were included. Genotypes of the three single nucleotide polymorphisms (SNPs) of *LOXL1* (rs1048661, rs3825942, and rs2165241) were analyzed by direct sequencing, and a case-control association study was performed.

**Results:**

The three SNPs were significantly associated with exfoliation syndrome (XFS) and exfoliation glaucoma (XFG) individually. After controlling for rs3825942 and rs2165241, the association between rs1048661 and XFS/XFG remained significant (p=3.6×10^-7^). At this SNP, the T allele and TT genotype conferred a 7.59-(95% confidence interval [CI]: 3.87–14.89, p=6.95×10^-11^) and 8.69-(95% CI: 4.15–18.20, p<1.00×10^-7^) fold increased risk to the disease. The alleles of T at rs1048661 and C at rs2165241 were found to be risk alleles in Chinese subjects, which were opposite to Caucasian individuals. The haplotypes T-G, defined by SNPs rs1048661 and rs3825942, and T-C by SNPs rs1048661 and rs2165241, were also significantly associated with the disorder. However when the genotypic or allelic frequencies of the three SNPs were compared between XFS and XFG, no significant difference was detected.

**Conclusions:**

*LOXL1* is a susceptibility gene of XFS/XFG in the Chinese population, and the association is mainly attributed to SNP rs1048661. The risk alleles of rs1048661 and rs2165241 in Chinese subjects were found to be opposite to that of Caucasians. The genotypic and allelic distributions of these SNPs are similar between XFS and XFG.

## Introduction

Exfoliation syndrome (XFS) is an age-related disorder of the extracellular matrix in which abnormal fibrillar material is produced and progressively accumulated in tissues throughout the anterior segment and also in the connective tissues of various viscera. The clinical features of this disorder, which was initially described by Lindberg in 1917 [1], are deposition of white flake-like material on the anterior lens surface, the pupillary border, trabecular meshwork, zonula, ciliary body, and other anterior segment structures. XFS is a common identifiable cause for secondary glaucoma, i.e., exfoliation glaucoma (XFG), which is characterized by rapid progression, high resistance to medical therapy, and poor prognosis. XFS can not only lead to severe chronic open-angle glaucoma but also to acceleration of cataract formation, lens subluxation, angle closure glaucoma, and severe complications at the time of cataract extraction, such as zonular dialysis, capsular rupture, and vitreous loss [1–4]. XFS is also a systemic disorder that is primarily related to vasculopathy, including transient ischemic attack, hypertension, angina, and myocardial infarction [1-4].

The prevalence of XFS varies greatly among ethnic groups, with a prevalence of 10–20% among the elderly populations of Finland [5,6], Iceland [7], Sweden [8], and Greece [9], while being 0% in Greenland Eskimos [10,11]. In Asian populations, the prevalence is relatively lower, being 3.01–6.28% in Indians aged over 40 years [12,13], 3.4% in Japanese aged over 50 years [14], 0.4% in Hong Kong Chinese aged over 60 years [15], and 0.2% and 0.7% in Singaporean Chinese aged over 40 and 60 years, respectively [16].

So far, the exact cause of the production of exfoliation material is still unknown, but there is evidence showing that genetic factors may play an important role in the pathogenesis of XFS [6,10,17–23]. Ethnic differences in the prevalence of XFS, positive family history of XFS, increased risk for XFS in relatives [6,18–20,22], and twin studies [17] support the inheritance of XFS. Multiple inheritance patterns have been suggested, but no clear pattern is evident, implying that XFS is a complex disorder [6,10,19,22,23]. A genome-wide linkage study has reported a promising genetic locus on 18q as well as other potential loci [24].

Recently, a genome-wide association study in the Icelandic and Swedish populations indicated that three single nucleotide polymorphisms (SNPs) in the lysyl oxidase-like 1 (*LOXL1*) gene on chromosomal region 15q24 were significantly associated with XFS and XFG [25]. The three SNPs are rs2165241 in the first intron and rs1048661 and rs3825942 in the first exon of the gene, which lead to substitution of amino acids at position 141 and 153, denoted as R141L and G153D, respectively [25]. LOXL1 is one of the five lysyl oxidase family members that catalyzes oxidative deamination of lysine residues of tropoelastin, which leads to the spontaneous cross-linking with consequential formation of elastin polymer fibers [25–27]. Studies showed that *LOXL* knockout mice have diffuse connective tissue-associated changes secondary to failed elastic fiber homeostasis [27–29]. XFS is proposed to arise from abnormal production and aggregation of elastin microfibrillar components produced by various cell types of the eye and other extraocular tissues [1,3,30]. Moreover, LOXL1 and elastin were found to be expressed in various ocular tissues, and *LOXL1* mRNA expression was differentially regulated dependent on the phase of progression of the fibrotic process and stages of XFS syndrome [30,31]. Therefore, the hypothesis that defects in *LOXL1* may cause XFS is biologically reasonable.

The association between XFS and the three *LOXL1* SNPs has been confirmed in other Caucasian [31–38] and Indian populations [39]. In the Japanese population, the SNPs rs1048661 and rs2165241 also showed association with XFS, but the risk alleles were opposite to the Caucasian study [40–45]. So far, however, the role of these SNPs in XFS has rarely been studied in the Chinese population except one study in Singaporean Chinese in which rs3825942 was found to be associated with the disorder but no association was found for rs1048661 [46]. Therefore, this study was conducted to investigate the association between the *LOXL1* variants and XFS in the Chinese population.

## Methods

### Study subjects

The diagnostic criterion for exfoliation syndrome is the existence of exfoliation material on the anterior lens capsule with dilation of the pupils or on the pupil margin in either eye. Patients with intraocular pressure (IOP) of less than 21 mmHg and no clinical evidence of glaucomatous optic neuropathy were classified as XFS. While exfoliation glaucoma (XFG) was diagnosed if the patient had the above characteristics of exfoliation syndrome and the following features: (1) IOP ≥22 mmHg in either eye; (2) glaucomatous changes on the optic disc, defined as cup to disc ratio >0.7 in either eye or an asymmetric cup to disc ratio of >0.2 or notching of the disc rim; and (3) characteristic glaucomatous visual field loss [47]. Cases with other causes for secondary glaucoma, such as uveitis, pigment dispersion syndrome, and iridocorneal endothelial syndrome, were excluded. The patients were recruited from the following five hospitals, which were located in or near Beijing: Beijing Tongren Hospital, XingTai Eye Hospital, Peking University Third Hospital, Handan Eye Hospital, and Anyang Eye Hospital. Controls were individuals randomly selected from a population-based healthy entity in which 6830 people were recruited in a previous, comprehensive, ophthalmic, epidemiologic study in a county in north China near Beijing [48]. The controls were enrolled by the following criteria: (1) having no signs of XFS or XFG, (2) no glaucomatous changes on optic disc, (3) normal visual field and intraocular pressure, (4) no family history of glaucoma, and (5) no other eye diseases except mild refractive errors. As exfoliation syndrome is a late-onset disorder and rarely develops before the age of 50 years, only individuals aged 50 years or above were included into this study as controls. All study subjects were unrelated Han Chinese. They received comprehensive ophthalmic examinations, including visual acuity testing and refraction, Goldmann applanation tonometry, gonioscopy, slit lamp biomicroscopy in mydriasis, fundus examination, and automated static perimetry (Humphrey Visual Field Analyzer; Carl Zeiss Ophthalmic Systems, Inc. Humphrey Division, Dublin, CA). Peripheral venous blood was obtained from each subject.

The research protocol was approved by the ethics committee for human research of Beijing Tongren Hospital, Capital Medical University in Beijing, China. Informed consent was obtained from all participants after explaining the objective and nature of the study. The study was conducted in accordance with the Declaration of Helsinki.

### Analysis of *LOXL1* polymorphisms

Genomic DNA was extracted from whole blood by using the Genomic DNA Extraction Kit, (Ebioshine Beijing Biotechnology Co., Ltd., Beijing, China). The three SNPs (rs1048661, rs3825942, and rs2165241) in the *LOXL1* gene, according to the previous report, were amplified by PCR and were directly sequenced [25]. Two sets of primers were used for amplification by PCR (Table 1). The PCR protocol was as follows: initial denaturation at 94 ^o^C/5 min, followed by 10 cycles (94 ^o^C/30 s, 65–60 ^o^C touchdown for 1 min with 0.5 ^o^C decrement, 72 ^o^C/45 s), 30 cycles (94 ^o^C /30 s, 60 ^o^C /1 min, 72 ^o^C /45 s), and a final extension at 72 ^o^C/1 min. Genotypes of the three *LOXL1* SNPs were determined by direct DNA sequencing, using BigDye Terminator v3.1 Kit (Applied Biosystems, Foster City, CA) in a 3730XL capillary sequencer (Applied Biosystems). The sequences were analyzed by Sequencing Analysis software v5.2 (Applied Biosystems).

**Table 1:** Primer sequences for PCR for SNPs of *LOXL1*.

**Amplicon**	**Primer sequences**	**Size (bp)**
rs1048661 and rs3825942*	F: 5’-CAACGGGCAGGTGTACAGCTT-3’	441
	R: 5’-GCGGGGTCGTAGTTCTCGTAC-3’	
rs2165241	F: 5’-CTCTAGGGCCCCTTGGAGAAT-3’	321
	R: 5’-GGCCAGAGGTCTGCTAAGCAC-3’	

Two sets of primers were used for PCR for the three SNPs. The asterisk indicates that the same set of primer was used for rs1048661 and rs3825942.

### Statistical analysis

Hardy–Weinberg equilibrium (HWE) was tested by using the χ^2^ test in SAS Genetics (v9.1, SAS Institute Inc., Cary, NC). The comparison of allelic and genotypic frequencies between case and control groups as well as haplotype association analysis was performed using a standard χ^2^ test with SAS Genetics. The Bonferroni method was used for the adjustment of multiple comparisons, with a p value of less than 0.017 (0.05/3) being considered as statistically different. Odds ratios (OR) with 95% confidence intervals (CI) were estimated by the SAS Genetics software and SPSS software (ver.12.0, SPSS inc., Chicago, IL). In order to exploit the major SNP that contribute to the association between *LOXL1* and the disorder, a conditional haplotype-based association test was performed using PLINK (v1.06, Shaun Purcell) [49].

The SNP that has independent haplotyic effect and explains the omnibus association is considered as the predominant SNP. The allelic and genotypic association analysis as well as haplotype association analysis were also cross-checked with PLINK. The allelic, genotypic and haplotype frequencies between two sub-phenotypes, i.e., XFS without glaucoma and XFG, were compared using the methods mentioned above. Pairwise linkage disequilibrium (LD) analysis among the three SNPs was performed in Haploview (ver. 4.0, Daly Lab, Broad Institute, Cambridge, MA). The sensitivity (SE), specificity (SP), positive and negative predictive values (PPV and NPV) of the at-risk alleles, and genotypes for the three SNPs were calculated to evaluate their abilities of predicting the affection status using the adjusted estimation methods [50] ( the prevalence of XFS was estimated to be 0.2% [16]).

## Results

A total of 50 patients with exfoliation syndrome, including 43 cases with XFG and seven cases with XFS without glaucoma, as well as 125 control individuals were recruited into this study. The age at recruitment was 54–87 years (mean age 70.4±7.6 years) in cases, 54–80 years (mean age 63.8±5.1 years) in controls. The gender distribution between cases and controls was not significantly different (p=0.593), with 31 (62.0%) males and 19 (38.0%) females in cases and 72 (57.6%) males in controls. The IOP of the controls was 9–20.25 mmHg (mean IOP 14.18±2.25 mmHg). The clinical features of the patients are shown in Table 2. Thirty out of the 43 XFG patients had severe glaucoma and had trabeculectomy more than once, and 20 cases were treated with medicine.

**Table 2:** The clinical features of the patients with XFS/XFG.

**Clinical features**	**Total (n=50)**		
	**XFG (n=42)**	**XFS with high IOP (n=1)**	**XFS (n=7)**
Age at recruitment (mean ± SD)	70.20±7.57	73	71.28±8.94
Range	(54-87)	(73-73)	(54-82)
Gender (male/female)	28/14	1/0	2/5
VCDR (mean ± SD)	0.78±0.15	0.3	0.40±0.08
History of trabeculectomy	n=30	n=0	n=0
History of laser trabeculoplasty	n=1	n=0	n=0
History of laser iridotomy	n=3	n=0	n=0
Treated with medicine	n=19	n=1	n=0

A total of 50 patients with exfoliation syndrome were recruited into this study, including 43 cases with XFG and 7 cases with XFS without glaucoma. Thirty out of the 43 XFG patients had trabeculectomy for more than once and 20 cases were treated with medicine.

Unequivocal genotypes of the three SNPs were obtained from both patients and controls. The genotype distribution of SNP rs1048661 deviated slightly from the HWE in controls (p=0.0245). SNPs rs3825942 and rs2165241 followed the HWE in controls (p=0.53 for rs3825942 and p=0.21 for rs2165241, respectively). We further checked the reproducibility of genotyping of rs1048661 by re-performing DNA sequencing. The results were in complete agreement with that obtained the first time. Therefore, non-concordance with HWE for this SNP is not due to genotyping errors.

Allelic association analysis showed that there are significant differences in the allelic distributions between the two groups for the three SNPs (Table 3). The T allele of rs1048661 was at a significantly higher frequency in cases than in controls (p=6.95×10^-11^, OR =7.59, 95% CI: 3.87–14.89). SNP rs3825942 was also associated with exfoliation syndrome (p=8.00×10^-4^), with the at-risk allele G presenting in 100% of patients. At rs2165241, the frequency of the C allele was significantly higher in cases than in controls (p=1.10×10^-2^, OR=5.44, 95% CI: 1.27–23.43). The genotypic frequencies for each of the three SNPs were also compared between cases and controls (Table 3). The frequency of genotype TT at SNP rs1048661 was significantly higher in cases than in controls (p<1.00×10^-7^, OR=8.69, 95% CI: 4.15–18.20), and the frequencies of GG and GT genotypes were significantly lower in cases than in controls. The genotypes of rs3825942 was associated with exfoliation syndrome (p=3.84×10^-3^), with genotype GG being found in all patients, while GA and AA were detected only in controls. At rs2165421, only two genotypes, i.e., CC and CT, were detected. The genotype CC presented at a significantly higher frequency in cases than in controls (p=8.00×10^-3^), conferring approximately sixfold increased risks to exfoliation syndrome (OR=6.0, 95% CI: 1.37–26.37). In the conditional haplotype-based association test, rs1048661 had an independent haplotypic effect after controlling for the other two SNPs (p=3.6×10^-7^), while rs3825942 (p=0.0147) and rs2165241 (p=0.498) did not have an independent haplotypic effect after controlling for the other two SNPs. Moreover, after controlling for rs1048661, the other two SNPs were no longer associated with the disorder (p=0.0462).

**Table 3:** Allele and genotype association analysis for the three SNPs of *LOXL1*.

**SNP**	**XFS/XFG (n=50)** **Count (proportion)**	**Control (n=125)** **Count (proportion)**	**x^2^**	**p value**	**OR (95%CI)**
rs1048661					
Allele					
T	89 (0.89)	129 (0.52)	42.53	6.95×10^-11^	7.59 (3.87-14.89)
G	11 (0.11)	121 (0.48)			
Genotype					
TT	43 (0.86)	27 (0.22)	67.72	<0.001	8.69 (4.15-18.20)**
GT	3 (0.06)	75 (0.60)			
GG	4 (0.08)	23 (0.18)			
Total	43/3/4 (TT/GT/GG)	27/75/23 (TT/GT/GG)	61.72	3.97×10^-15^	NA
rs3825942					
Allele					
G	100 (1.00)	224 (0.90)	11.24	8.00×10^-4^	NA*
A	0 (0.00)	26 (0.10)			
Genotype					
GG	50 (1.00)	101 (0.80)	11.13	3.84×10^-3^	NA*
GA	0 (0.00)	22 (0.18)			
AA	0 (0.00)	2 (0.02)			
Total	50/0/0 (GG/GA/AA)	101/22/2 (GG/GA/AA)	17.63	3.84×10^-3^	NA
rs2165241					
Allele					
C	98 (0.98)	225 (0.90)	6.42	0.01	5.44 (1.27-23.43)
T	2 (0.02)	25 (0.10)			
Genotype					
CC	48 (0.96)	100 (0.80)	7.01	8.00×10^-3^	6.0 (1.37-26.37)**
CT	2 (0.04)	25 (0.20)			
TT	0 (0.00)	0 (0.00)			
Total	48/2/0 (CC/CT/TT)	100/25/0 (CC/CT/TT)	7.01	8.00×10^-3^	NA

There were significant differences for the allelic proportion between case and control groups for all the three SNPs. T allele of rs1048661, G of rs3825942, and C of rs2165241 were risk alleles for the disorder. The genotypes TT for rs1048661, GG for rs3825942, and CC for rs2165241 were “risk” genotypes for the disease. Total indicate the general test of association in the 2-by-3 table of disease-by-genotype. NA: not available. The single asterisks indicate that all patients had G allele and genotype of GG for rs3825942, so the OR values couldn’t be calculated. The double asterisks indicate the OR values and p values derived from comparision of the specific genotype with all the others, i.e., TT versus GT+GG at rs1048661, CC versus CT+TT at rs2165241.

**Figure 1:**
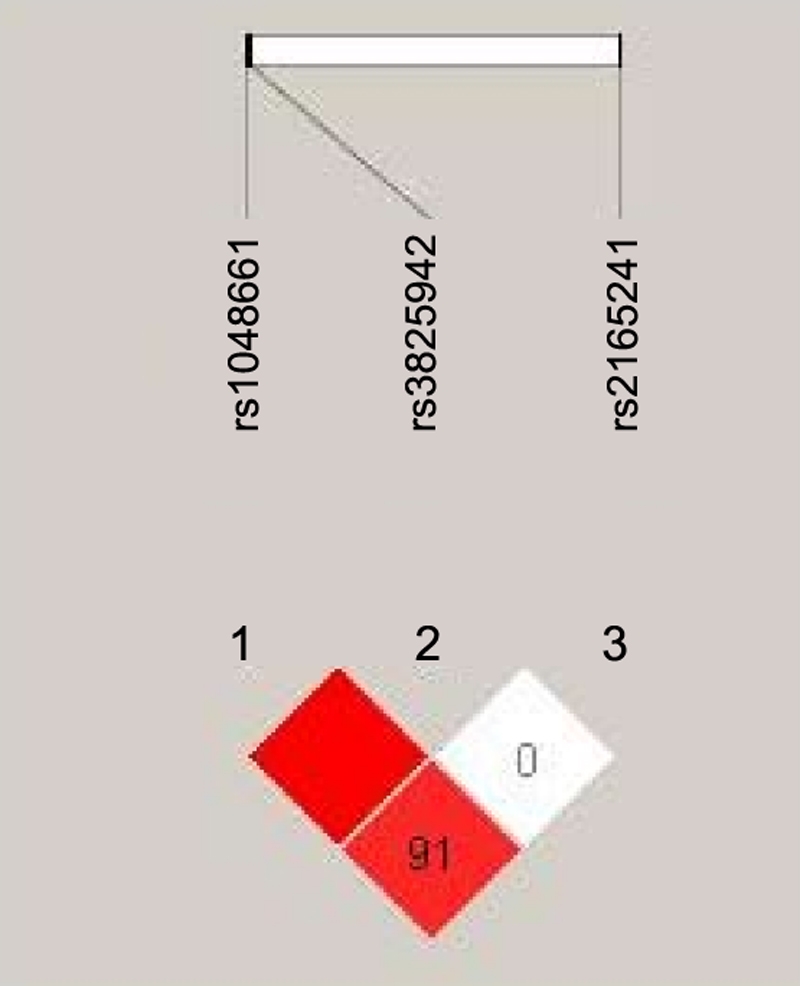
Analysis of linkage disequilibrium (LD) among the three SNPs of *LOXL1*. The SNPs rs1048661 and rs3825942, and rs1048661 and rs2165241 were in strong LD, but rs3825942 and rs2165241 were not in LD. The numbers “91” and “0” in the check indicate 100×D’ (D’ means coefficient of linkage disequilibrium).

Pairwise LD analysis showed that SNPs rs1048661 and rs3825942 were in strong LD (Coefficient of linkage disequilibrium [D’] =1.000, LOD score [LOD] =6.96), SNPs rs1048661 and rs2165241 were also in LD (D’=0.913, LOD=5.14), but rs3825942 and rs2165241 were not in LD (D’=0.007, LOD=0.00; Figure 1). Pairwise SNPs with an LOD score of  >3 were considered to be in LD [51].

Haplotypes defined by the three SNPs were analyzed (Table 4). For the SNPs rs1048661 and rs3825942, three haplotypes were observed. The haplotype T-G was identified to be significantly associated with exfoliation syndrome, conferring an approximately sevenfold increased risk to the disease (OR=6.77, 95% CI: 2.69–17.03, p<1.00×10^-3^). The haplotype G-A was observed only in controls. For the SNPs rs1048661 and rs2165241, four haplotypes were observed, among which the haplotype T-C was significantly associated with an increased susceptibility of XFS (OR=6.99, 95% CI: 2.78–17.58, p<1.00×10^-3^). For the SNPs rs3825942 and rs2165241, three haplotypes were observed, with the haplotype G-C being at a significantly higher frequency in cases than in controls (p=0.002, OR=12.18, 95% CI: 1.61–92.09). The haplotype A-C was only observed in controls. The haplotypes for the three SNPs were also estimated. The haplotype T-G-C was at a significantly higher frequency in cases than in controls (p<1.00×10^-3^, OR=6.77, 95% CI: 2.69–17.03). Besides, the haplotype T-G-T was observed only in cases, while the haplotypes G-A-C and G-A-T were observed in controls exclusively.

**Table 4:** Haplotype Association analysis between the *LOXL1* SNPs and XFS/XFG

Haplotype			Proportion		x^2^	p value	OR (95%CI)
			Case	Control			
rs1048661	rs3825942						
T	G		0.89	0.516	22.09	<1.00×10^-3^	6.77 (2.69 - 17.03)
G	G		0.11	0.38	11.67	0.001	0.22 (0.09 - 0.55)
G	A		0	0.104	4.21	0.04	NA *
Total			NA	NA	20.36	<1.00×10^-3^	NA
rs1048661	rs2165241						
T	C		0.88	0.516	20.47	<1.00×10^-3^	6.99 (2.78 - 17.58)
G	T		0.01	0.1	8.12	0.004	0.08 (0.01 - 0.62 )
T	T		0.01	0	2.52	0.112	NA *
G	C		0.1	0.384	13.64	<1.00×10^-3^	0.18 (0.07 - 0.48)
Total			NA	NA	19.70	<1.00×10^-3^	NA
rs3825942	rs2165241						
G	C		0.98	0.8	9.15	0.002	12.18 (1.61 - 92.09)
G	T		0.02	0.1	1.95	0.163	NA *
A	C		0	0.1	9.159	0.002	0.001(0 - inf)
Total			NA	NA	13.45	0.001	NA
rs1048661	rs3825942	rs2165241					
T	G	C	0.88	0.516	19.70	<1.00×10^-3^	6.77 (2.69 - 17.03)
G	G	C	0.1	0.29	7.04	0.008	0.28 (0.10 - 0.75)
G	G	T	0.01	0.09	2.59	0.108	NA
T	G	T	0.01	0	2.51	0.113	NA *
G	A	C	0	0.094	3.76	0.052	NA *
G	A	T	0	0.01	0.40	0.526	NA *
Total			NA	NA	20.37	<1.00×10^-3^	NA

The haplotypes T-G, T-C and G-C for the SNPs rs1048661 and rs3825942, rs1048661 and rs2165241, and rs3825942 and rs2165241 were identified to be significantly associated with XFS, respectively. The haplotype T-G-C for the three SNPs was identified to be significantly associated with XFS. Total indicate omnibus tests, while the others indicate the haplotype-specific tests. The OR values were obtained from comparision of each individual haplotype with all the other haplotypes, i.e., T-G versus G-G + G-A for the SNPs rs1048661 and rs3825942, et al. NA: not available. The asterisks indicate that the OR values were not available since differences between cases and controls were not significant for these haplotypes (p>0.017).

The at-risk alleles and genotypes of the three SNPs were analyzed for their abilities to predict the affection status (Table 5). High SE (87.7%, 95% CI: 78.6–96.8%) but low SP (19.8%, 95% CI: 12.8–26.8%) was found for the T allele of rs1048661. The TT genotype of this SNP was with increased SP (77.1%, 95% CI: 69.7–84.5%) and high SE (82.3%, 95% CI: 71.7–92.9%). Although the NPVs were high, the PPVs were low for the risk alleles and genotypes of all three SNPs.

**Table 5:** The sensitivity, specificity, positive and negative predictive values for the risk alleles and genotypes of the three SNPs of *LOXL1*.

**SNP**	**SE (95%CI)**	**SP (95%CI)**	**PPV (95%CI)**	**NPV (95%CI)**
rs1048661				
allele (T)	0.877(0.786-0.968)	0.198(0.128-0.268)	0.0022(0.0019-0.0025)	0.9988(0.9988-1.00)
genotype (TT)	0.823(0.717-0.929)	0.771(0.697-0.845)	0.0072(0.0047-0.0097)	0.9995(0.9992-0.9998)
rs3825942				
allele (G)	0.948(0.886-1.00)	0.037(0.004-0.070)	0.0020(0.0019-0.0021)	0.9972(0.9930-1.00)
genotype (GG)	0.948(0.886-1.00)	0.206(0.135-0.277)	0.0024(0.0021-0.0027)	0.9995(0.9989-1.00)
rs2165241				
allele (C)	0.948(0.886-1.00)	0.022(0.004-0.048)	0.0019(0.0017-0.0020)	0.9953(0.9875-1.00)
genotype (CC)	0.912(0.833-0.991)	0.213(0.141-0.285)	0.0023(0.0020-0.0026)	0.9992(0.9984-1.00)

SE, SP, PPV and NPV indicate sensitivity, specificity, positive and negative predictive values, respectively. High SE (82.3%) and high SP (77.1%) were found at the TT genotype of rs1048661.

The allelic, genotypic, and haplotypic frequencies of the three SNPs between the two sub-phenotypes, i.e., XFS without glaucoma and XFG, were not significantly different (p>0.05, data not shown).

## Discussion

The association between the *LOXL1* gene and XFS/XFG in the Chinese population has rarely been reported except in one recent report from Singapore [46]. In this present study, three major *LOXL1* SNPs were found to be significantly associated with XFS/XFG, even though the sample size was as small as 50. Hence, the effect sizes of these SNPs in the Chinese populaton were large, and 50 samples provided a good statistical power to detect significant association. In the Chinese population, however, it is usually difficult to recruit a large sample of XFS/XFG because of the low disease prevalence (0.2–0.7%) in this ethnic group [15,16]. The reason for the low prevalence in Chinese individuals is still unknown. Similarly, the prevalence of this disorder is also relatively low in Japanese and other Asian populations compared with the Caucasian populations [12–14]. Such a discrepancy in disease prevalence might not be fully explained by the ethnic difference in the frequencies of the at-risk alleles at the *LOXL1* SNPs (Table 6). Hence, other genetic and/or environmental factors, yet to be identified, might be involved in the development of the disorder.

**Table 6:** Risk alleles and MAF for the three SNPs of *LOXL1* in different populations.

**Population**	rs1048661 ** (G/T)**		rs3825942 ** (G/A)**		rs2165241 ** (T/C)**		**Reference**
	**Risk allele**	**MAF**	**Risk allele**	**MAF**	**Risk allele**	**MAF**	
Iceland	G	0.349 (T)	G	0.153 (A)	T	0.473 (T)	[25]
Sweden	G	0.318 (T)	G	0.121 (A)	T	0.465 (C)	
Austria	G	0.329 (T)	G	0.183 (A)	NA	NA	[36]
United States	G	0.335 (T)	G	0.156 (A)	T	0.487 (T)	[34]
United States	G	0.297 (T)	G	0.202 (A)	T	0.448 (T)	[32]
United States	G	0.400 (T)	G	0.120 (A)	NA	NA	[33]
United States	G	0.281 (T)	G	0.205 (A)	T	0.456 (T)	[35]
Germany and Italy	G	0.348 (T)	G	0.149 (A)	T	0.488 (T)	[37]
Australia	G	NA	G	NA	NA	NA	[31]
refSNP (European)	NA	0.040 (T)	NA	0.172 (T)	NA	0.392(T)	NCBI Database
India	*	0.270 (T)	G	0.070 (A)	NA	NA	[39]
Japan	T	0.450 (G)	G	0.147 (A)	NA	NA	[41]
Japan	T	0.497 (G)	G	0.137 (A)	C	0.102 (T)	[43]
Japan	T	0.460 (G)	G	0.143 (A)	NA	NA	[40]
Japan	T	NA	G	NA	NA	NA	[45]
Japan	T	0.493 (T)	G	0.123 (A)	NA	NA	[42]
Japan	T	0.450 (T)	G	0.194 (A)	C	0.124 (T)	[44]
Singapore (Chinese)	*	0.444 (G)	G	0.082 (A)	NA	NA	[46]
China (Beijing)	T	0.484 (G)	G	0.104 (A)	C	0.100 (T)	present study
China							[52]
(Hongkong)	NA	0.470 (G)	NA	0.124 (A)	NA	0.102 (T)	
(Beijing)	NA	0.497 (G)	NA	0.135 (A)	NA	0.084 (T)	
refSNP							NCBI Database
Asian (China)	NA	NA	NA	0.111(T)	NA	0.067 (T)	
Asian (Japan)	NA	0.438 (G)	NA	0.125 (T)	NA	0.167 (T)	

NA indicates not available. The asterisk indicates that no association was found. MAF indicates minor allele frequency.

In this present study, the association between the three *LOXL1* SNPs and XFS /XFG has been replicated in the Chinese cases. However, like the findings reported from the Japanese population [40–45], the risk alleles at SNPs rs1048661 and rs2165241 were different from that in Caucasian populations, as shown in Table 6. The risk allele T at SNP rs1048661 was found to confer a 7.6-fold increased risk to XFS/XFG (95% CI: 3.87–14.89, p=6.95×10^-11^), while the homozygous genotype TT was found to have an even larger effect size, with an OR of 8.69 (95% CI: 4.15–18.20, p<1.00×10^-7^). The reasons for the discrepancy in the genotypic distributions of this SNP among XFS/XFG patients with different ethnicities remain unknown. Conditional haplotype-based association test showed that rs1048661 had independent haplotyic effect (p=3.6×10^-7^), and the association between the other two SNPs and the disorder was no longer significant after controlling for rs1048661 (p=0.0462), suggesting that this SNP is the major SNP contributing to the association. Moreover, in one study in an Indian population and one study in a Singaporean Chinese population, no association was found for rs1048661 with the disorder [39,46]. In an American study, the rs1048661 was not associated with the disorder after controlling the other two SNPs [35]. These findings suggest that there are other yet-to-be-identified modifier factors (environmental or genetic) among different populations involved in the pathogenesis of XFS/XFG. Since the association profiles of *LOXL1* and XFS/XFG were distinctively different between Beijing Chinese and Singaporean Chinese cases [46], further investigations of this association from multiple centers are needed to unravel the role of *LOXL1* in the genetics of XFS/XFG among the Chinese population.

The SNP rs1048661 is located in the first exon of the *LOXL1* gene and leads to a substitution of amino acids at position 141 [25]. The effect of such a substitution on *LOXL1* mRNA expression has been investigated in order to provide clues on the role of this SNP in the pathogenesis of XFS/XFG. Thorleifsson and colleagues observed a decrease of 7.7% in *LOXL1* mRNA expression in adipose tissue of Caucasian patients with the G allele [25], whereas Kazuhiko and colleagues did not observe any change of *LOXL1* mRNA expression in lens capsule from Japanese XFG patients [45]. On the other hand, irrespective of individual genotype, ocular *LOXL1* expression was found to be differentially regulated in different stages of the disorder. The *LOXL1* mRNA expression was upregulated in the early stage but was decreased in an advanced stage [30]. Further studies are needed to clarify the role of this SNP in the expression of *LOXL1* and subsequently in the pathogenesis of this disorder.

Besides rs1048661, SNPs rs3825942 and rs2165241 were also significantly associated with XFS/XFG individually. However, when controlled for rs1048661, the association became insignificant, suggesting they are more likely to be genetic markers in LD with the rs1048661. The rs3825942 was associated with the disorder in Caucasians [25,35] and Japanese [43]. However, this SNP has no functional impact on the *LOXL1* mRNA level in adipose tissue [25] or anterior lens capsule [45]. Concerning the SNP rs2165241, which is located in the intronic region, the functional impact of this SNP remains unknown. Similar to SNP rs1048661, the association profile of rs2165241 resembles that in a Japanese population [43,44]  but was opposite to the Caucasians [25,34].

In this present study, although the sample size of XFS without glaucoma was small (n=7), the allelic and genotypic frequencies of the three SNPs in the two sub-phenotypes were similar and had no statistical significance. This finding is consistent with other studies [25]. The *LOXL1* gene is more likely a susceptibility gene for XFS rather than a triggering factor for the development of XFG, and there may be other genetic or environmental factors predisposing individuals toward XFG.

High SE and SP for the risk genotype TT at SNP rs1048661 (82.3%, 95% CI: 71.7–92.9% and 77.1%, 95% CI: 69.7–84.5%) imply the potential value of the diagnostic test for XFS, although further studies are needed. However applying genotyping of the SNPs was inappropriate in screening patients with XFS among this Chinese population due to low PPVs (Table 5).

In summary, we confirmed the results that the three SNPs of *LOXL1* are associated with XFS/XFG, but the risk alleles at rs1048661 and rs2165241 in this present study are opposite to Caucasian populations. The rs1048661 is the predominant SNP associated with XFS/XFG in our study cohort. The role of this SNP in pathogenesis of the disorder needs to be studied further, and further investigations are also needed to unravel additional genetic or environmental factors modifying the development of this disorder.
